# The Role of Claudin-1 in Enhancing Pancreatic Cancer Aggressiveness and Drug Resistance via Metabolic Pathway Modulation

**DOI:** 10.3390/cancers17091469

**Published:** 2025-04-27

**Authors:** Daisuke Kyuno, Hinae Asano, Reona Okumura, Kumi Takasawa, Akira Takasawa, Takumi Konno, Yuna Nakamori, Kazufumi Magara, Yusuke Ono, Masafumi Imamura, Yasutoshi Kimura, Takashi Kojima, Makoto Osanai

**Affiliations:** 1 Department of Pathology, Sapporo Medical University School of Medicine, Sapporo 060-8556, Japan; 2 Department of Surgery, Division of Gastroenterological Surgery, Sapporo Medical University, Sapporo 060-8556, Japan; 3 Division of Tumor Pathology, Department of Pathology, Asahikawa Medical University, Asahikawa 078-8510, Japan; 4 Department of Cell Science, Institute of Cancer Research, Sapporo Medical University School of Medicine, Sapporo 060-8556, Japan

**Keywords:** aldo-keto reductase, claudin-1, chemoresistance, pancreatic cancer, tight junction

## Abstract

Background/Objectives: Pancreatic ductal adenocarcinoma is a lethal malignancy, necessitating an understanding of its molecular mechanisms for the development of new therapeutic strategies. The tight junction protein claudin-1, known to influence cellular functions in various cancers and is considered a therapeutic target, remains unclear in pancreatic cancer. Methods: This study assessed claudin-1 expression in resected pancreatic cancer samples, public databases, and pancreatic cancer cell lines. *Claudin-1* knockout with CRISPR/Cas9 on poorly differentiated pancreatic cancer cell lines and a proteome analysis were performed to investigate the intracellular mechanisms of claudin-1. Results: Claudin-1 was markedly overexpressed in pancreatic ductal adenocarcinoma and intraepithelial neoplasia compared to normal ducts, and high claudin-1 levels were an independent predictor of poor prognosis. *Claudin-1* knockout diminished cell proliferation, migration, invasion, and chemoresistance in pancreatic ductal adenocarcinoma. Proteome analysis revealed the significant downregulation of aldo-keto reductase family proteins (AKR1C2, AKR1C3, and AKR1B1) in *claudin-1* knockout cells, which are linked to metabolic pathways. Aldo-keto reductase knockdown reduced chemoresistance, proliferation, and invasion in these cell lines. Conclusions: These findings indicate that the abnormal expression of claudin-1 promotes tumor progression and drug resistance through its interaction with aldo-keto reductase proteins, highlighting claudin-1 and aldo-keto reductase family proteins as potential biomarkers and therapeutic targets for pancreatic cancer.

## Simple Summary

Pancreatic ductal adenocarcinoma is a lethal malignancy, requiring an understanding of its molecular mechanisms for new therapies. Claudin-1, a tight junction protein influencing cellular functions in various cancers and considered a therapeutic target, has an unclear role in pancreatic cancer. This study assessed claudin-1 expression in resected pancreatic cancer samples, public databases, and cell lines. Claudin-1 was overexpressed in pancreatic ductal adenocarcinoma and intraepithelial neoplasia compared to normal ducts, and high levels predicted poor prognosis. Claudin-1 knockout reduced cell proliferation, migration, invasion, and chemoresistance in pancreatic ductal adenocarcinoma. A proteome analysis revealed significant downregulation of aldo-keto reductase family proteins in claudin-1 knockout cells linked to metabolic pathways. Aldo-keto reductase knockdown reduced chemoresistance, proliferation, and invasion in these cell lines. These findings indicate that abnormal claudin-1 expression promotes tumor progression and drug resistance through interaction with aldo-keto reductase proteins, highlighting both as potential biomarkers and therapeutic targets in pancreatic cancer.

## 1. Introduction

Pancreatic ductal adenocarcinoma (PDAC) is a highly lethal malignancy, characterized by an extremely low 5-year survival rate. Although there has been a modest increase in this survival rate over the past few decades, from 0.9% in 1975 to 4.2% in 2011 across all stages, survival remains poor [1]. Recent estimates suggest a slight improvement, with studies reporting survival rates of 13% in the USA between 2013 and 2019 [2] and 12.1% in Japan in 2015 [3]. Advances in surgical techniques and systemic chemotherapy have led to 5-year survival rates approaching 30–40% following resection and adjuvant therapy [4,5]. Moreover, early diagnosis has contributed to improved survival outcomes, with the 5-year survival rate for Stage IA PDAC reported at 83.7% in 2012 [6]. However, for patients who do not undergo resection, the 5-year survival rate remains unchanged and alarmingly low, ranging from 0.8% to 0.9% [1]. PDAC is typically diagnosed at an advanced stage due to the absence of early symptoms and effective screening methods. Over 75% of patients with PDAC present with locally advanced or metastatic disease, and more than 95% either have metastases at diagnosis or develop them during follow-up [7]. Understanding the molecular mechanisms involved in PDAC invasion and metastasis is crucial for developing novel therapeutic strategies.

Tight junction proteins are vital for maintaining the integrity and function of epithelial and endothelial barriers [8]. These proteins are integral components of tight junctions, which are specialized structures that seal spaces between adjacent cells. Traditionally, tight junction proteins have been viewed as tumor suppressors that inhibit cell growth and proliferation. However, recent studies have shown that these proteins can also exhibit tumor-promoting effects. For instance, junctional adhesion molecule A enhances the proliferation and migration of breast cancer cells by interacting with L-type amino acid transporter 1 to facilitate amino acid uptake [9]. Additionally, a variant of cingulin promotes cancer progression by inducing epithelial–mesenchymal transition (EMT) [10]. Claudin-2 (Cldn2) has been implicated in promoting liver metastasis in breast and colorectal cancers [11,12], while claudin-6 (Cldn6) is associated with chemotherapy resistance and poor prognosis in gastric and cervical adenocarcinomas [13,14]. In pancreatic cancer cells, the localization of the tight junction protein tricellulin promotes cell proliferation and invasiveness through its association with the MAPK and PKC pathways [15]. These findings suggest that tight junction proteins can promote tumorigenesis under certain conditions, thereby contributing to cancer progression [16]. To identify new molecular targets for therapy, it is essential to analyze the functions of tight junction proteins in tumor cells and elucidate the mechanisms underlying their tumor-promoting activities.

Claudin-1 (Cldn1), a critical component of tight junctions, plays a key role in maintaining cellular polarity, regulating paracellular transport, and facilitating cell–cell communication [17]. In cancers, its role is complex, with studies demonstrating its tumor-promoting and -suppressing effects [18,19]. This dual role varies depending on specific cellular processes, the expression levels of Cldn1, and the cancer type. However, the specific functions of Cldn1 and its clinical significance in pancreatic cancer cells remain poorly understood [20,21]. Thus, investigating the precise role of Cldn1 in pancreatic cancer could aid in identifying new therapeutic targets and improving prognosis.

In this study, we aimed to elucidate the role of Cldn1 in pancreatic cancer by generating *Cldn1*-knockout (KO) clones using CRISPR/Cas9 and analyzing their cellular dynamics.

## 2. Materials and Methods

### 2.1. Antibodies

The primary and secondary antibodies utilized in this study are listed in Supplementary Table S1.

### 2.2. Immunohistochemical Analysis of Surgical Specimens and Immunocytochemistry of Cell Blocks

Specimens from 77 PDAC cases obtained through surgical resection between 2011 and 2016 were retrieved from the pathology files of Sapporo Medical University Hospital. This study received approval from the Institutional Review Board of Sapporo Medical University (IRB study number 292-68). Clinical information, including perioperative chemotherapy regimens, was obtained from the medical records. At our institution, the standard approach for resectable (R) and borderline resectable (BR) PDAC during the study period consisted of upfront surgery followed by six months of adjuvant chemotherapy. Neoadjuvant therapy was introduced for BR-PDAC in 2013 and for R-PDAC in 2019. Adjuvant therapy was administered to all patients after resection, with gemcitabine as the first-line agent between 2006 and 2012. After 2013, S-1 (tegafur/gimeracil/oteracil potassium) was adopted as the first-line adjuvant treatment.

As controls, adjacent non-neoplastic regions were examined as normal tissues (*n* = 63). The 77 cases were staged according to the Union for International Cancer Control (UICC) staging system (8th edition) [22]. The clinicopathological features of the patients were assessed based on the general rules for the study of pancreatic cancer, 8th edition (in Japanese) [23]. Immunohistochemistry was performed using anti-Cldn1 (1:100), dihydrodiol dehydrogenase (DD) (1:200), and aldo-keto reductase 1B1 (AKR1B1) (1:100) antibodies. DD antibodies recognize AKR1C isoforms of AKR1C1, -C2, -C3, and -C4. As the trends of AKR1C2 and AKR1C3 were the same in the pancreatic cancer cells used in this study, DD antibodies that depict both were used in this experiment. Tissue sections were deparaffinized in xylene, rehydrated using a graded series of ethanol and phosphate-buffered saline (PBS), and incubated with 3% hydrogen peroxide (H_2_O_2_) for 10 min to block endogenous peroxidase activity. After antigen retrieval through microwave heating at 95 °C for 30 min in 10 mmol/L Tris/1 mmol/L EDTA buffer, the sections were incubated overnight at 4 °C with antibodies diluted in Dako REAL™ Antibody Diluent (Agilent, Santa Clara, CA, USA). The sections were then incubated with the Dako REAL™ EnVision™ Detection System for 30 min at room temperature, followed by color development using the Dako REAL™ EnVision™ Detection System (DAB) for 3–5 min as the chromogen, according to the manufacturer’s instructions. The slides were counterstained with hematoxylin.

The staining intensity of the surgical specimens was scored as follows: 0, no reactivity in the cell membrane or cytoplasm; 1+, faint/almost no reactivity; 2+, weak-to-moderate reactivity; 3+, moderate-to-strong reactivity. The proportion of the stained area was scored between 0 and 100 in 10% increments based on the percentage of stained cells. A histological score (H-score) ranging from 0 to 300 was calculated by multiplying the proportion and intensity scores. The H-scores of PDAC and normal pancreatic ductal epithelial cells are presented as box plots overlaid with dot plots. For slide evaluation, two pathologists and two observers (DK, HA, RO, and MO) were blinded to the clinical data. Discordant cases were discussed, and a consensus on the H-score was reached.

For the immunocytochemistry of cell blocks, cells were harvested from culture dishes using a cell lifter and collected via centrifugation at 300× *g* for 3 min. The cell pellets were fixed in formalin overnight at 4 °C, followed by paraffin embedding and sectioning. Immunostaining was performed as previously described.

### 2.3. Cell Culture and Treatment

The human pancreatic cancer cell line PK45H was obtained from the RIKEN Bio-Resource Center. The cells were maintained in RPMI-1640 with L-glutamine (NACALAI TESQUE, Kyoto, Japan) supplemented with 10% fetal bovine serum (FBS) (Corning, Corning, NY, USA), 100 U/mL penicillin, and 100 μg/mL streptomycin (Penicillin–Streptomycin; Thermo Fisher Scientific, Waltham, MA, USA). The cell line was confirmed not to have mycoplasma contamination. All cells were plated on collagen-coated 35 and 60 mm culture dishes and incubated in a humidified 5% CO2 incubator at 37 °C. *Claudin-1* knockout (*Cldn1*-KO) cell lines were generated using the CRISPR/Cas9 system with the GeneArt CRISPR Nuclease Vector Kit (Thermo Fisher Scientific), following the manufacturer’s instructions. Briefly, the guide RNA (gRNA) target site was identified using CRISPR Direct software (https://crispr.dbcls.jp/ (accessed on 1 December 2017)) [24]. The sequence of the target site in the human *Cldn1* locus was 5′-CAACAGCTGCAGCCCCGCGTTGG-3′. A vector expressing non-coding gRNA, orange fluorescent protein (OFP), and Cas9 nuclease was constructed using the GeneArt CRISPR Nuclease Vector Kit (Thermo Fisher Scientific). Cells were transfected with the vector using Lipofectamine 3000 (Thermo Fisher Scientific). Two days after transfection, OFP-positive cells were sorted using FACS AriaTM III (BD Biosciences, Franklin Lakes, NJ, USA). The loss of Cldn1 protein expression was confirmed using Western blotting.

In experiments with anticancer drugs, cell viability and half maximal inhibitory concentration (IC50) were assessed using the WST-8 assay or cell-counting analysis 48 h after treatment with the following drugs: etoposide (100 μM, referenced by [25,26]), doxorubicin (5 μM), and daunorubicin (5 μM).

### 2.4. Western Blotting

Cells were scraped from 60 or 35 mm dishes using 500–300 μL of buffer containing 1 mM sodium bicarbonate (NaHCO_3_) and 2 mM phenylmethanesulfonyl fluoride, collected in microcentrifuge tubes and sonicated for 10 s. Cell lysates were quantified using a BCA Protein Assay Kit (Thermo Fisher Scientific) and SkanIt RE software (version 7.0.2, Thermo Fisher Scientific). Protein samples (8 μg/lane) were separated via electrophoresis on 5–20% sodium dodecyl sulfate–polyacrylamide gels (Wako, Osaka, Japan) and transferred to nitrocellulose membranes (Immobilon; Millipore, Bedford, UK). The membranes were washed three times for 5 min at room temperature with washing buffer and incubated with horseradish peroxidase-conjugated anti-mouse and anti-rabbit IgG antibodies at room temperature for 1 h. Immunoreactive bands were visualized using enhanced chemiluminescence (Bio-Rad Laboratories, Hercules, CA, USA). All images were captured using Lumino Graph II (Atto, Tokyo, Japan).

### 2.5. Cell Proliferation and Plate Colony Formation Assay

For cell counting in the proliferation assay, cell numbers were assessed using the TC20TM Automated Cell Counter (Bio-Rad Laboratories, Hercules, CA, USA) at 24, 48, and 72 h after seeding in 24-well plates (5000 cells/well). For the WST-8 assay, cells were seeded in 96-well plates (5000 cells/well), and their viability was assessed at 24, 48, and 72 h after incubation using the Cell Counting Kit-8 (Dojindo Laboratories, Kumamoto, Japan), following the manufacturer’s instructions. Absorbance at 450 nm was measured using a Varioskan LUX spectrophotometer (Thermo Fisher Scientific).

For the colony-formation assay, cells were seeded in 6-well plates (2000 cells/well) and incubated for 10–14 days. The cells were fixed with ice-cold methanol for 10 min and stained with 0.04% crystal violet solution for 30 min at room temperature. Cell clusters of at least 50 cells were defined as positive colonies. Visible colonies were counted using ImageJ software Version 1.54p 17 February 2025 (National Institutes of Health) [27].

### 2.6. Wound-Healing, Migration, and Invasion Assays

For the wound-healing assay, cells were plated in 24-well plates. Confluent cell sheets were scratched with 200 µL pipette tips to generate straight-line gaps. Each gap was marked with a line to ensure consistent field acquisition during image capture at 24, 48, and 72 h after incubation. The scratched distance was measured using ImageJ software.

Migration and invasion assays were performed using Transwell chambers (8 μm pore polycarbonate membrane insert; Corning) and BioCoat Matrigel invasion chambers (#354480; Corning, Corning, NY, USA), respectively, in 24-well plates. Cells in medium without FBS were plated onto Transwell and Matrigel chambers for 48 h. The lower chamber of the Transwell and Matrigel chambers was filled with a medium containing 10% FBS. At 48 h after plating, migrating and invading cells were fixed with ethanol for 5 min and visualized using 0.04% crystal violet solution. The areas of migrating and invading cells were measured using ImageJ software. Each experiment was repeated independently three times.

### 2.7. Proteome Analysis

Proteome analysis was carried out as described previously [14]. Gene Ontology (GO) term enrichment and Kyoto Encyclopedia of Genes and Genome (KEGG) pathway analyses were conducted on DAVID (https://david.ncifcrf.gov/ (accessed on 15 May 2023)) [28,29].

### 2.8. Immunocytochemistry

Cells were grown on 35 mm glass-bottom dishes (Iwaki, Chiba, Japan) and incubated for 1 day. They were then fixed with cold acetone and ethanol (1:1) at −20 °C for 10 min. After rinsing with PBS, the cells were incubated with primary antibodies at 4 °C overnight. The following day, the cells were stained with DyLight^®^ 488 (green)-conjugated anti-rabbit IgG and DyLight^®^ 594 (red)-conjugated anti-mouse IgG antibodies (1:200) at room temperature for 1 h. The nuclei were counterstained with 4′,6-diamidino-2-phenylindole (DAPI). The stained cells were observed under a fluorescence microscope (Olympus, Tokyo, Japan). Co-localization analysis was performed using the ImageJ colocalization plugin, coloc 2. Pearson’s R value showed the degree of correlation, and Costes *p*-value of 1.00 indicated >95% certainty that colocalization existed [30].

### 2.9. Cellular Fractionation and Co-Immunoprecipitation Assays

Cellular fractionation was performed using a Cell Fractionation Kit (#9038; Cell Signaling Technology, Danvers, MA, USA) according to the manufacturer’s instructions. Co-immunoprecipitation assay was performed using the Capturem™ IP & CO-IP kit (Takara-Bio, Otsu, Japan) according to the manufacturer’s protocol. The proteins were immunoprecipitated using anti-Cldn1 polyclonal and isotype control antibodies (5 ug in each sample, 20 min, room temperature). Quantitative analysis was performed using the ImageJ software and the Band/Peak Quantification tool [31].

### 2.10. RNA Interference

Cells were transfected with universal negative control siRNAs (Merck, Darmstadt, Germany) and gene-specific siRNAs using Lipofectamine™ RNAiMAX (Thermo Fisher Scientific) to knock down *AKR1C2*, *AKR1C3*, and *AKR1B1*, following the manufacturer’s instructions. Two siRNAs were used to knock down each gene, and the results for each knockdown are presented separately in the Figures and Supplementary Figures. The siRNA sequences used are listed in Supplementary Table S2.

### 2.11. Apoptosis Assay

Apoptosis induced by anticancer drugs was evaluated using flow cytometry with a Muse^®^ Caspase-3/7 kit (Cytek Biosciences, Fremont, CA, USA) 24 h after treatment, following the manufacturer’s instructions. Daunorubicin-treated cells were excluded from this assay because their autofluorescence considerably influenced the results [32,33].

### 2.12. Statistical Analysis

Data are expressed as the average ± standard deviation. The analysis was conducted using unpaired two-tailed Student’s *t*-test, Fisher’s exact test, and the Mann–Whitney U test. Survival rates were determined through the Kaplan–Meier method and compared using the log-rank test and the Cox proportional hazards model. Univariate and multivariate regression analyses were performed using the Cox proportional hazards model to estimate the hazard ratios and 95% confidence intervals. Statistical significance was set at *p* < 0.05. All statistical analyses were performed using BellCurve for Excel version 4.07 and EZR software version 1.68 [34].

### 2.13. Ethics Statement and Patient Consent

This study was reviewed and approved by the Sapporo Medical University Ethics Committee (approval number: 292-68). The study was approved on 25 August 2017. All the patients provided informed consent to participate in the study.

Informed Consent: The need for informed consent was waived by the institutional review board of Sapporo Medical University because of the retrospective nature of the study.

Registry and the Registration No. of the study/trial: N/A.

Animal Studies: N/A.

## 3. Results

### 3.1. Expression Profiles of Cldn1 in Surgical Specimens of PDAC, PanIN, and Normal Ducts

**Figure 1:**
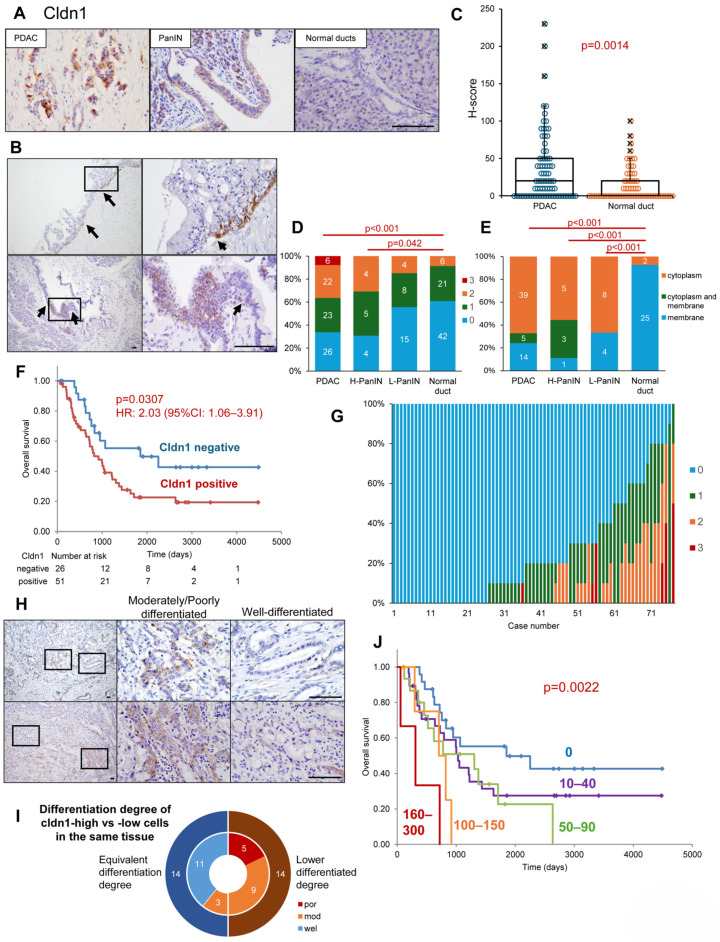
(**A**): Immunohistochemistry of Cldn1 in surgical specimens of PDAC, PanIN, and normal pancreatic ducts. (**B**) Immunohistochemistry of Cldn1 at the border between normal ductal epithelial cells and PanIN. The squares in the left panel indicate the locations of the right panels. Arrows indicate the border. (**C**) The H-score of Cldn1 in PDAC and normal ducts. The crosses indicate outliers. (**D**) The expression intensities of Cldn1 in PDAC, H- and L-PanIN, and normal ducts. Numbers in the bars indicate the number of cases. (**E**) Location of Cldn1 in PDAC, H- and L-grade PanINs, and normal ducts. (**F**) Kaplan–Meier curves for overall survival of patients with PDAC divided by the presence or absence of Cldn1 expression in cancer cells. (**G**) The overall expression intensity of Cldn1 in PDAC tissues. (**H**) Immunohistochemistry of Cldn1 in the PDAC tissues. These two rows show two separate cases of well-differentiated and moderately/poorly differentiated adenocarcinomas in the same field of view. The squares in the left panel indicate the locations of the middle and right panels. (**I**) Comparison of differentiation degrees between high- and low-Cldn1 expression cells in the same cancer tissue. The inner pie chart shows the differentiation degree of Cldn1-high cells. (**J**) Kaplan–Meier curves for the overall survival of patients with PDAC divided by the H-score of Cldn1. Bar: 100 um. PDAC, pancreatic ductal adenocarcinoma; Cldn1, claudin-1; H-score, histological score.

We examined Cldn1 expression and distribution in PDAC, pancreatic intraepithelial neoplasia (PanIN), and normal pancreatic ducts using resected PDAC specimens. Cldn1 was strongly detected in PDAC, moderately detected in PanIN and weakly or not expressed in normal ducts (Figure 1A and Figure S1A). In the continuous area between normal pancreatic ductal epithelial cells and PanIN, Cldn1 was detected in PanIN with a boundary (Figure 1B). The H-score of Cldn1 was significantly higher in PDAC than in the normal pancreatic ducts (*p* = 0.0014; Figure 1C). The proportion of highly expressed Cldn1 (intensity of 2+ or 3+) was significantly greater in PDAC and H-PanIN than in normal pancreatic ducts (*p* < 0.001 and *p* = 0.042, respectively; Figure 1D). Cldn1 was on the plasma membrane of normal pancreatic ductal epithelial cells, whereas it was expressed in the cytoplasm of most PanIN and PDAC cells (Figure 1A and Figure S1A). The expression sites of Cldn1 were significantly altered in L- and H-PanIN and pancreatic cancer cells compared to those in normal ductal cells (*p* < 0.001, *p* < 0.001, and *p* < 0.001, respectively; Figure 1E).

Using the database of our hospital, we performed a survival analysis of patients with PDAC regarding Cldn1 expression. There was no significant correlation between Cldn1 positivity and the clinicopathological features (Table S3). The patients were divided into two groups based on their H-scores: Cldn1-positive (H-score > 0) and Cldn1-negative (H-score = 0). Fifty-one (66.2%) of 77 PDAC cases showed Cldn1 expression, and the overall survival was significantly shorter in the Cldn1-positive group (Figure 1F). Similarly, in public databases, overall survival and disease-free survival were significantly lower in the Cldn1-high expression group (Human Protein Atlas (https://www.proteinatlas.org/ (accessed on 1 February 2025)), Kaplan–Meier Plotter (https://pancreas.kmplot.com/ (accessed on 1 February 2025)) (Figure S1B, Table S4) [35,36]. The Cox proportional hazards model revealed that Cldn1 expression, pStage, and high postoperative carbohydrate antigen 19-9 were independent predictors of overall survival (Table 1). Furthermore, Cldn1 was highly expressed in the metastatic lymph nodes and neural invasion areas of pancreatic cancer cells (Figure S1C).

The differentiation degree was examined for each Cldn1 expression intensity. A detailed observation of Cldn1 of pancreatic cancer cells in resected specimens revealed that the intensity of Cldn1 expression was not homogeneous within the same pancreatic cancer tissue (Figure 1G). Within the same tissue, there was a trend toward Cldn1 expression in intermediate-to-poorly differentiated pancreatic cancers, even when Cldn1 expression was weak or absent in well-differentiated pancreatic cancers (Figure 1H). In half of the cases, cancer cells with high Cldn1 expression (intensity score 3-2) were less differentiated than those with low Cldn1 expression (intensity score 1-0) in the same tissue (Figure 1I). Additionally, the cancer cells in the invasive front were highly expressed Cldn1 compared with the cells in the center of the tumor (Figure S1D). When patients were grouped by H-score, the H-score and overall survival were inversely related (Figure 1J and Figure S1E). The immunostaining of these pancreatic cancer tissues revealed that Cldn1 was highly expressed in the cytoplasm of some cancer cells and that Cldn1 was associated with a poor differentiation of cancer cells and poor prognosis in patients with PDAC.

### 3.2. Cldn1 Contributes to the Proliferation, Migration, and Invasion of Pancreatic Cancer Cells

**Figure 2:**
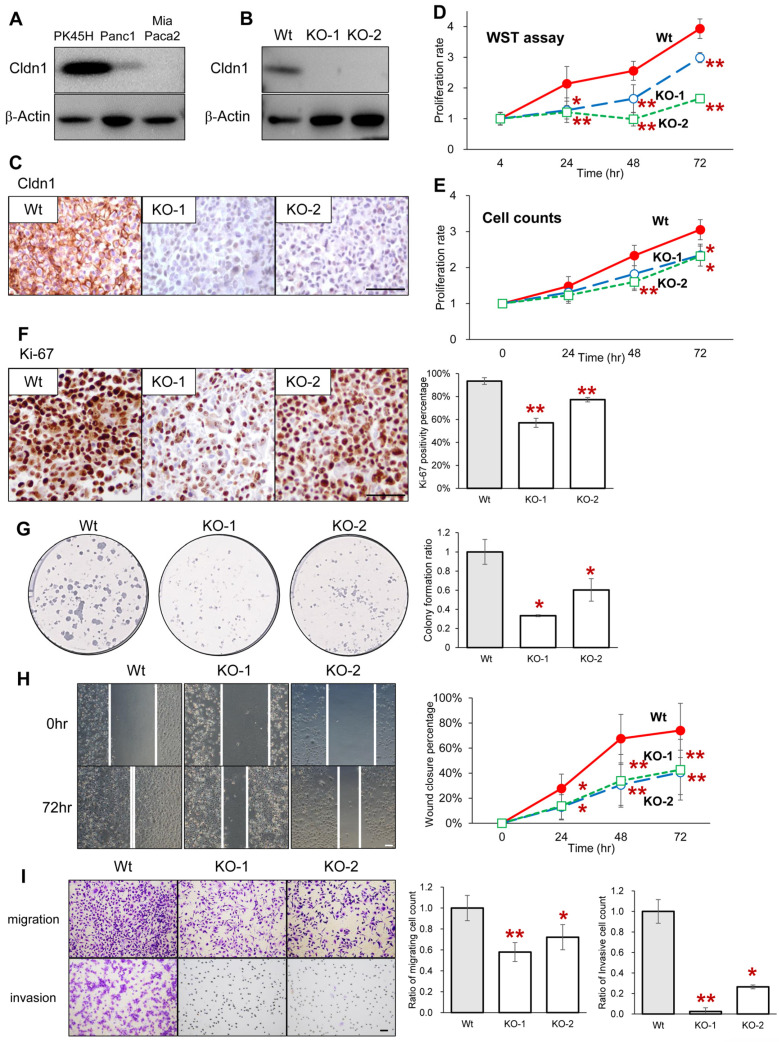
(**A**) Western blotting for Cldn1 in poorly differentiated pancreatic adenocarcinoma cell lines. (**B**,**C**) Western blotting (**B**) and immunohistochemistry (**C**) of Cldn1 in Wt and *Cldn1*-KO clones of PK45H cells (**C**) D and E: WST-8 (**D**) and proliferation assays through the cell counting (**E**) of Wt and *Cldn1*-KO clones. (**F**–**I**): Immunohistochemistry of Ki-67 (**F**), colony formation assay (**G**), wound healing assay (**H**), and migration and invasion assays using a double chamber of Wt and *Cldn1*-KO clones (**I**). Right-side graphs show the quantification of each assay. Bar: 100 μm. Graphs represent mean ± standard deviation (SD). * *p* < 0.05 vs. Wt, ** *p* < 0.01 vs. Wt. Wild type, Wt; Knockout, KO.

To examine the significance of Cldn1 expression in pancreatic cancer cells for malignancy, we established PK45H cells, which are poorly differentiated pancreatic cancer cells, utilizing a CRISPR/Cas9-based method for the stable knockout of Cldn1. Western blot analysis revealed that PK45H cells exhibited the highest levels of Cldn1 among the poorly differentiated pancreatic cancer cell lines (Figure 2A). Both Western blotting and immunohistochemistry of cell blocks verified the absence of Cldn1 expression in Cldn1-KO cells (Figure 2B,C). The WST-8 cell proliferation assay and the cell counts indicated that the knockout of Cldn1 significantly reduced the proliferation of PK45H cells (Figure 2D,E). The *Cldn1*-KO cells showed a significant decrease in the number of Ki-67-positive cells (Figure 2F). The colony formation assay showed that the number of colonies of *Cldn1*-KO cells was significantly lower than that of wild-type cells (Figure 2G). Scratched and double chamber assays were performed to investigate the relationship between Cldn1 and migration and invasion. After 24, 48, and 72 h, the distance of wound closure was significantly shorter in *Cldn1*-KO cells than in wild-type cells (Figure 2H). The double chamber assay showed that *Cldn1*-KO cells had fewer migratory and invasive abilities (Figure 2I). These indicate that Cldn1 contributes to the proliferation, migration, and invasiveness of pancreatic cancer cells.

### 3.3. Comprehensive Proteome Analysis Showed AKRs as Candidates That Interact with Cldn1 in Pancreatic Cancer Cells

**Figure 3:**
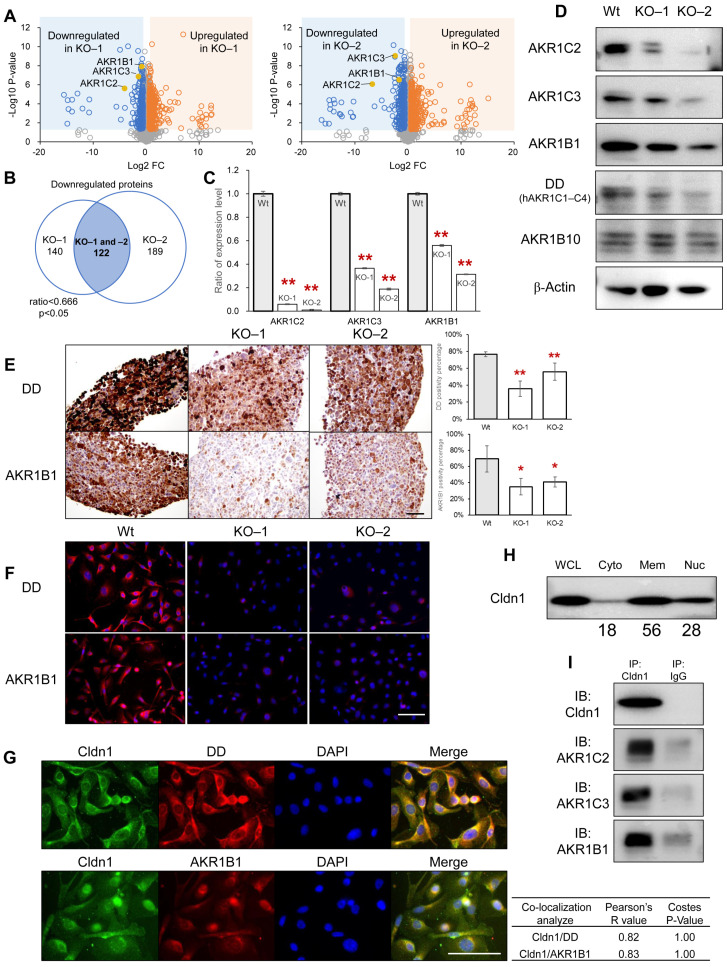
(**A**): Volcano plots of proteins identified through mass spectrometry-based proteomic analysis of Wt and *Cldn1*-KO cells. Blue dots indicate significantly decreased proteins (ratio < 0.666, *p* < 0.05), and orange dots indicate significantly increased proteins (ratio > 1.5, *p* < 0.05) in *Cldn1*-KO clones compared with those in the wild type. The left plots compare Wt and KO-1 clones, while the right plots compare Wt and KO-2 clones. AKR1C2, AKR1C3, and AKR1B1 were downregulated in both plots (yellow dots). (**B**) Venn map of downregulated proteins in KO-1 and KO-2 cells. (**C**–**F**) AKR1 protein expression, as revealed through mass spectrometry-based proteomic analysis (**C**), Western blotting (**D**), immunohistochemistry (**E**), and immunofluorescence (**F**), and in Wt and *Cldn1*-KO cells. DD is an alternative name for human AKR1C proteins. The right-side graph shows the quantification of the assay (**G**). The top row shows immunofluorescence of Cldn1 (green), DD (red), and DAPI (blue), and the bottom row shows immunofluorescence of Cldn1 (green), AKR1B1 (red), and DAPI (blue) in PK45H Wt cells. The table shows the results of colocalization analysis. (**H**) Cellular fractionation assay of Cldn1 in PK45H Wt cells showed that 18% and 28% of Cldn1 was detected in the cytoplasmic and cytoskeletal/nuclear fractions, respectively. The values below the bands represent densitometry intensity ratios. (**I**) Interaction of Cldn1 with AKR1C2, -C3, and -B1 proteins was examined using co-immunoprecipitation assay. Bar: 100 μm. Graphs represent mean ± SD. * *p* < 0.05 vs. Wt, ** *p* < 0.01 vs. Wt. Cldn1, claudin-1; AKR, aldo-keto reductase; DD, dihydrodiol dehydrogenase; DAPI, 4,6-diamidino-2-phenylindole; Wt, wild type; KO, knockout; WCL, whole cell lysate; Cyto, cytoplasm, Mem, membrane; Nuc, nuclear.

To clarify the molecular mechanisms behind the Cldn1-dependent phenotypes mentioned earlier, we conducted an extensive shotgun proteome analysis. This proteomic approach was employed to contrast the protein expression profiles between *Cldn1*-KO and wild-type cells. To ensure data accuracy and reliability, each sample underwent independent analysis four times (Data S1). We identified a total of 3763 unique proteins. Among these, 122 were found to be downregulated (ratio < 0.666, *p* < 0.05), and 208 were upregulated (ratio > 1.5, *p* < 0.05) in *Cldn1*-KO-1 and -2 cells compared to wild-type cells (Figure 3A,B). We performed GO and KEGG pathway analyses of downregulated and upregulated proteins (Tables S5 and S6). In the KEGG pathway analysis, metabolic pathways (hsa01100) were identified with statistical significance for downregulated proteins. Proteins in the metabolic pathways included the AKR superfamily: AKR1C2, AKR1C3, and aldose reductase (AKR1B1) (Table 2). These proteins are upregulated in parallel with the aberrant expression of cldn6 in cervical adenocarcinoma and increased anticancer drug resistance in cancer cells [14]. In this study, the expression levels of the AKR superfamily proteins were lower in cld1-KO cells than in wild-type cells (Figure 3A,C). The decreased expression levels of AKR superfamily proteins in *Cldn1*-KO cells were also confirmed through Western blotting, immunofluorescence, and immunohistochemistry using anti-AKR1B1 and DD antibodies, which recognize AKR1C isoforms of AKR1C1, -C2, -C3, and -C4. (Figure 3D–F). Proportionality between *Cldn1* and *AKR1C2*, *-C3*, and *-B1* has also been shown in public databases (Figure S2A: cBioPortal; https://www.cbioportal.org/ (accessed on 24 July 2023)) [37,38]. In PK45H wild-type cells, immunofluorescence, co-localization analysis, co-immunoprecipitation, and cellular fractionation assays demonstrated that Cldn1 was expressed in the cytoplasm and around the nucleus, and that DD and AKR1B1 were at the same site, overlapping Cldn1 (Figure 3G–I).

These suggest that Cldn1 and AKR superfamily proteins are localized within the cytoplasm of PK45H cells and that *Cldn1* KO decreased the AKR superfamily proteins.

### 3.4. Cldn1 KO and AKR Superfamily Knockdown Attenuate Drug Resistance

**Figure 4:**
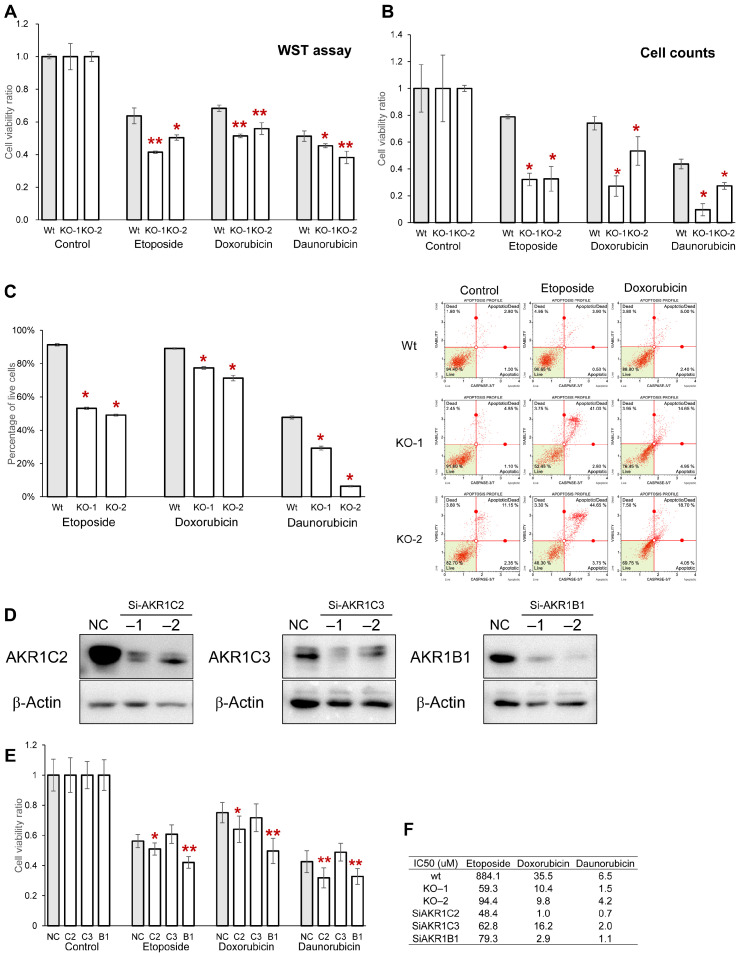
(**A**,**B**) WST-8 assay (**A**) and cell counting (**B**) 48 h after administering etoposide, doxorubicin, and daunorubicin. (**C**) Flow cytometry dot plots (**right**) and quantification (**left**) of apoptotic status based on Caspase-3/7 activation after administering etoposide, doxorubicin, and daunorubicin. (**D**) Western blotting of PK45H cells transfected with siRNA specific for AKR1 proteins. Two siRNAs were used for each gene. (**E**) WST-8 assay of PK45H cells transfected with siRNAs specific for *AKR1C2*, *AKR1C3*, and *AKR1B1* and NC after administering etoposide, doxorubicin, and daunorubicin. (**F**) IC50 values of PK45H Wt, *Cldn1* KO clones, and *AKR1C2*, *AKR1C3*, and *AKR1B1* knockdown clones for etoposide, doxorubicin, and daunorubicin. Graphs represent mean ± SD. * *p* < 0.05 vs. Wt or NC, ** *p* < 0.01 vs. Wt or NC. AKR, aldo-keto reductase; Wt, wild type; KO, knockout; NC, negative control.

Because the AKR superfamily is involved in anticancer drug resistance in cancer cells [14,39], the viability of cancer cells was assessed after exposure to etoposide, doxorubicin, and daunorubicin to clarify the effect of Cldn1 expression on drug resistance. *Cldn1*-KO cells showed significantly reduced resistance to these drugs compared with wild-type cells (Figure 4A,B). Flow cytometry revealed that *Cldn1* KO increased the proportion of cells that underwent apoptosis after exposure to etoposide and doxorubicin (Figure 4C). Daunorubicin-treated cells were excluded from the assay because of their autofluorescence.

To determine whether the decrease in anticancer drug resistance of the *Cldn1*-KO clones was owing to downregulation of the AKR superfamily, the *AKR1C2*, *-C3*, and *-B1* of PK45H cells were knocked down using siRNAs and examined for anticancer drug resistance. AKR1C2, -C3, and -B1 proteins were knocked down using two siRNAs (Figure 4D), and *AKR1C2* and *-B1* knockdown significantly attenuated the viability of cancer cells after exposure to etoposide, doxorubicin, and daunorubicin compared with that of the negative control (Figure 4E and Figure S2B). *AKR1C3* knockdown did not affect the resistance to anticancer drugs. The enhanced effects of antitumor drugs on Cldn1 knockout clones and AKR1C2, AKR1C3, and AKR1B1 knockdown clones were confirmed by their lower IC50 values compared to those of the wild type (Figure 4F). These results indicate that *Cldn1* KO and *AKR1C2* and *AKR1B1* knockdown increased the sensitivity of PK45H cells to anticancer drugs.

### 3.5. Knockdown of the AKR Superfamily Also Attenuates the Proliferation and Invasion Abilities of PK45H Cells

**Figure 5:**
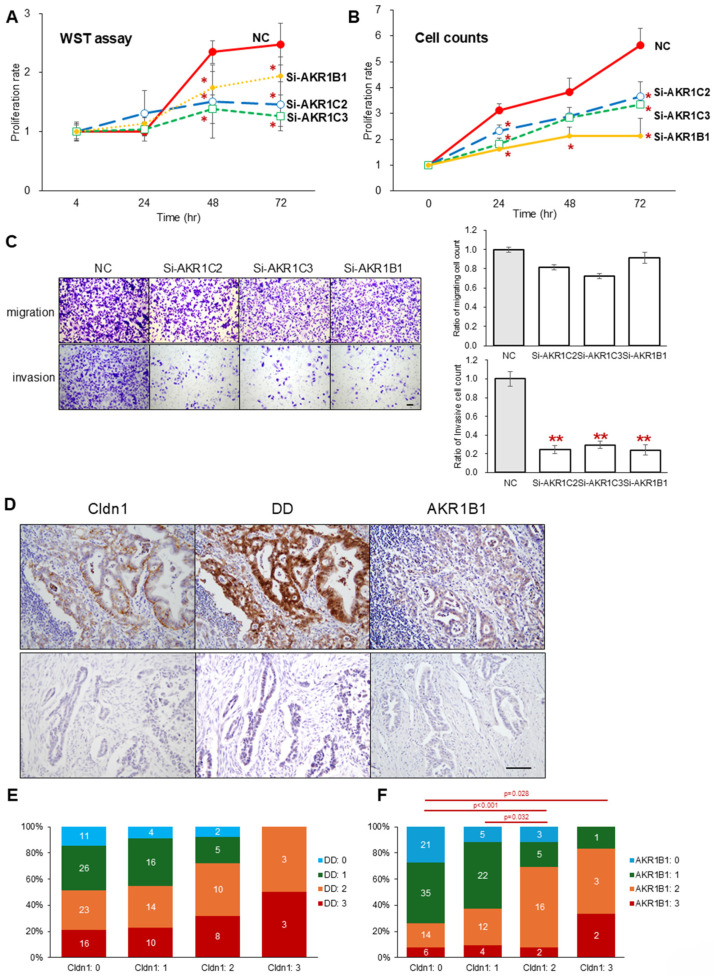
(**A**,**B**) WST-8 (**A**) and proliferation assays through cell counting (**B**) of NC and cells transfected with siRNAs specific for *AKR1C2*, *AKR1C3*, and *AKR1B1*. (**C**) Migration and invasion assays using double chamber (**left**) and quantification (**right**). There was no difference in the number of migrating cells between the knockdown and negative control groups; however, there was a significant decrease in the number of infiltrating cells in the knockdown group. (**D**) Immunohistochemistry for Cldn1, DD, and AKR1B1 in the same region. In the upper panel, all proteins are expressed in the cancer cells, whereas in the lower panel, no proteins are expressed. (**E**,**F**) Correlation between the expression intensity of Cldn1, DD (**E**), and AKR1B1 (**F**). Cldn1 and DD/AKRB1 expressions showed a proportional trend. Bar: 100 μm. Graphs represent mean ± SD. * *p* < 0.05 vs. NC, ** *p* < 0.01 vs. NC. Cldn1, claudin-1; AKR, aldo-keto reductase; wild type, Wt; knockout, KO; negative control, NC.

To investigate the significance of AKR superfamily downregulation in cancer cells, other effects of *AKR* superfamily knockdown in PK45H cells were assessed. The WST-8 cell proliferation assay and cell counts showed that *AKR1C2*, *-C3*, and *-B1* knockdown significantly inhibited the proliferation of PK45H cells (Figure 5A,B and Figure S2C). Scratch cell and double chamber assays showed that *AKR* superfamily knockdown did not affect migration ability. However, it significantly decreased the invasive ability of cancer cells compared with that of the negative control (Figure 5C and Figure S2D,E). Finally, the relationship between Cldn1 and the AKR superfamily was examined for its expression in surgical specimens of PDAC. Cancer cells expressing Cldn1 also expressed DD and AKR1B1 (Figure 5D and Figure S2F). Immunohistochemistry confirmed a proportional relationship between the Cldn1, DD, and AKR1B1 intensity scores in pancreatic cancer cells (Figure 5D–F).

## 4. Discussion

This study provides significant insights into the role of Cldn1 in PDAC and its potential as a therapeutic target. Our findings demonstrate that Cldn1 is overexpressed in PDAC tissues compared to normal pancreatic ducts and that its expression correlates with poorer patient prognosis. *Cldn1* KO in pancreatic cancer cell lines caused reduced cell proliferation, migration, and invasion, highlighting its role in promoting malignancy in pancreatic cancer cells.

One of the primary findings of this study was the differential expression of Cldn1 in PDAC and normal pancreatic tissues. Cldn1 was significantly overexpressed in PDAC, with a notable shift in its cellular localization from the membrane in normal ductal cells to the cytoplasm in PDAC cells. This alteration in Cldn1 localization may be indicative of its functional switch from a structural tight junction protein to a participant in tumor-promoting processes such as EMT, which is consistent with the findings of previous studies showing the involvement of claudin proteins in cancer progression [17,40,41]. The correlation between high Cldn1 expression and poor overall survival further underscores its potential role as a prognostic marker in PDAC, which is consistent with studies in other cancer types where Cldn1 has been associated with tumor aggressiveness and metastasis [42,43,44,45,46,47]. In studies of head and neck squamous cell carcinoma, increased Cldn1 expression has been associated with tumor progression through the inhibition of AMP-activated protein kinase activity and the activation of tumor growth factor-β signaling, which promotes tumor growth [48]. Similarly, in colon cancer cells, Cldn1 overexpression reportedly promotes tumor metastasis through the activation of Src and β-catenin signaling [49]. Recent reports have shown that Cldn1 is highly expressed in human lung adenocarcinoma-derived A549 cells and is involved in the augmentation of chemoresistance by the amino acid barrier [50]. In small cell lung cancer, overexpressed Cldn1 causes epithelial–mesenchymal transition, resulting in enhanced migration and reduced resistance to doxorubicin treatment [51]. Overexpression of CLDN1 promotes epithelial–mesenchymal transition, metastasis, resistance to anoikis, and chemotherapy in colon cancer cells through a direct interaction with ephrin type-A receptor 2 tyrosine kinase [42]. These findings indicate that Cldn1 overexpression induces cancer progression and metastasis and decreases patient prognosis in several cancer cell types. The mechanism by which Cldn1 expression is increased in pancreatic cancer cells remains unclear. Although it has been previously reported that TNF-α increases Cldn1 expression [52] and tumor growth factor-β and DNA methylation decrease it [41,53], other intracellular and extracellular mechanisms may also regulate Cldn1 expression. If such mechanisms can be clarified, it may be possible to reduce the aggressiveness of pancreatic cancer cells via the regulation of Cldn1.

Our findings contradict those of a recent study, which reported that Cldn1 loss might promote pancreatic cancer progression [53]. This study suggests that Cldn1 loss could be associated with more aggressive cancer behavior, in contrast to our results showing that Cldn1 expression correlates with poor prognosis and enhanced malignancy in pancreatic cancer cells. However, a survival analysis using information from public databases, such as The Cancer Genome Atlas, European Genome-phenome Archive, and Gene Expression Omnibus, supports our findings, revealing that patients with high *Cldn1* gene expression exhibit significantly lower survival rates than those with low *Cldn1* expression [35,36]. This discrepancy underscores the complexity of the Cldn1 role in pancreatic cancer. The expression of tight junction molecules, including claudins, is lower in poorly differentiated pancreatic cancer cells than in normal cells. This downregulation is usually associated with the promotion of EMT, a crucial process in cancer progression [41]. However, we observed high Cldn1 expression in a subset of pancreatic cancer cells, particularly in those with aggressive characteristics. According to the data we examined, only PK45H was a poorly differentiated pancreatic cancer cell line overexpressing Cldn1. Therefore, only one cell line, PK45H, could be experimentally examined for the effects of endogenous Cldn1 overexpression. The generalization of the experimental results from only one cancer cell line should be carefully considered. However, similar to the experimental results, an analysis using clinical and real-world data showed that Cldn1 expression was an unfavorable prognostic factor for patients with PDAC, and a correlation existed between Cldn1 and AKR family protein expression. In breast cancer, the expression, localization, and function of Cldn1 differ according to the molecular subtypes of the cancer [17,54], suggesting that Cldn1 may function differently depending on the cellular context and differentiation status. These inconsistent findings highlight the need for further research to clarify the precise role of Cldn1 in pancreatic cancer prognosis. Understanding the molecular mechanisms by which Cldn1 influences pancreatic cancer progression could provide critical insights into its potential as a therapeutic target. A deeper investigation into the specific functions and regulatory pathways of Cldn1 in pancreatic cancer is essential to resolve these conflicting observations and develop targeted therapies that could improve patient outcomes.

Our data suggest that Cldn1 contributes to the malignant behavior of PDAC cells through its interaction with the AKR family AKR1C2, AKR1C3, and AKR1B1 proteins. Proteome analysis revealed a significant downregulation of these proteins in *Cldn1*-KO cells, and subsequent functional assays demonstrated that this downregulation corresponded to reduced cancer cell proliferation, invasion, and drug resistance. AKRs are a superfamily of enzymes that are crucial in various cellular processes, including xenobiotic, steroid, and carbohydrate metabolisms [55]. The AKR family is overexpressed in several cancer types and has been implicated in cancer cell survival and chemoresistance, primarily through its role in the metabolism of chemotherapeutic agents and reactive aldehydes [39,56,57,58]. AKR1C2 induces chemotherapy resistance in bladder, lung, and gynecological cancers [59,60,61] and enhances the invasive and metastatic potential in esophageal and liver cancers [62,63]. AKR1C3 also induces chemotherapy resistance and aggressiveness in the cancer cells of hormone-dependent organs, such as the breast and prostate [64,65,66]. AKR1B1 overexpression causes tumor progression via several signaling pathways in cancer cells. AKR1B1 promotes the tumorigenicity and metastasis of triple-negative breast cancer [67] and the invasiveness of cervical and gastric cancers [68,69]. In pancreatic cancer, AKR1B1, upregulated by the β2-adrenergic receptor, promotes proliferation and inhibits apoptosis [70].

In this study, *Cldn1*-KO cells decreased resistance to chemotherapeutic agents such as etoposide, doxorubicin, and daunorubicin. These findings indicate that Cldn1 may play a critical role in modulating the efficacy of chemotherapeutic treatment for PDAC, possibly through its interaction with AKR family proteins. Notably, the previously reported IC50 values for doxorubicin in MIA PaCa-2 and PANC-1 cells, which exhibit low or undetectable CLDN1 expression, are 0.08 ± 0.03 µM and 0.07 ± 0.03 µM, respectively [71]. These values were markedly lower than the IC50 for PK45H wild-type cells in our data (35.5 µM), suggesting a potential link between high CLDN1 expression and increased resistance to chemotherapeutic agents. Aldo-keto reductases function as carbonyl reductases that catalyze the conversion of anthracyclines (doxorubicin and daunorubicin) into less-cytotoxic metabolites [72]. This enzymatic reaction decreases the intracellular levels of active drugs, thereby diminishing DNA damage and contributing directly to therapeutic resistance. In addition, AKR enzymes detoxify cytotoxic aldehydes generated during oxidative stress, thereby reducing reactive oxygen species-induced cellular damage caused by DNA-damaging agents, such as anthracyclines and etoposide [39]. This antioxidant function indirectly contributes to chemotherapy resistance by improving cancer cell survival during exposure to DNA-damaging agents. These findings suggest that AKR proteins contribute to resistance to DNA-damaging chemotherapeutics through direct enzymatic drug inactivation and indirect reduction of drug-induced oxidative stress. This relationship was further supported by our experiments, which showed that *AKR1C2 and AKR1B1* knockdown sensitized cancer cells to these drugs, whereas AKR1C3 knockdown had no significant effect. Furthermore, *AKR1C2*, *AKR1C3*, and *AKR1B1* knockdown attenuated the proliferation and invasive ability of cancer cells. The specific roles of these proteins in mediating chemoresistance and malignant behavior may provide novel targets for therapeutic intervention, potentially through the combined targeting of Cldn1 and AKR family members. Further research is needed to elucidate the precise regulatory mechanisms involving Cldn1 and AKRs in different cancer types and investigate potential therapeutic strategies targeting this pathway.

Several studies have suggested a relationship between the AKRs and tight junction proteins. Increased Cldn6 expression in cervical adenocarcinoma cells increases AKR family proteins and resistance to anticancer drugs [14]. Another study showed that AKR1B1 inhibition suppressed TGFβ1-induced airway remodeling and inhibited changes in occludin and E-cadherin levels in airway epithelial cells [73]. Owing to the limited number of reports on the association between AKRs and tight junction proteins, the detailed mechanisms remain unclear. Cldn1 depletion may trigger alterations in signaling pathways or intracellular trafficking dynamics that indirectly influence AKR gene transcription and mRNA stability. Loss of cldn1 alters the localization and transcriptional activity of ZONAB, consequently modifying downstream gene expression profiles [74]. Such claudin-dependent signaling changes could influence transcription factor activity or transcriptional cofactor recruitment at AKR1C promoters [75,76], leading to decreased AKR transcription. Alternatively, Cldn1 depletion may influence post-transcriptional regulatory mechanisms, including RNA-binding protein activity and altered miRNA expression profiles. Several miRNAs target AKR transcripts [77,78]. Changes in Cldn1 and the subsequent downstream signaling are associated with the differential expression of miRNAs or RNA-binding proteins [79], thereby affecting the mRNA stability or translational efficiency of the target genes. Such indirect transcriptional or post-transcriptional regulation may account for the reduced expression of AKR family proteins. In the present study, Cldn1 expression was correlated with AKR expression, but the study did not directly show how the molecules interact, and the regulatory mechanisms involving AKRs are diverse and require further detailed analysis of the associated intracellular signaling and transcription factor activity [39]. If these findings can be elucidated in future studies, they may provide a new therapeutic tool for controlling cancer cells. Our findings expand on this knowledge by suggesting that Cldn1 may regulate the expression of AKR1 proteins in PDAC cells. This mechanism provides a novel understanding of how Cldn1 might support the survival and aggressiveness of pancreatic cancer cells.

## 5. Conclusions

This study establishes Cldn1 as a crucial contributor to the malignancy of pancreatic cancer through its involvement in cellular proliferation, migration, invasion, and chemoresistance. The interaction between Cldn1 and AKR family proteins represents a novel mechanism that may be exploited for therapeutic purposes. Targeting Cldn1 alone or in combination with AKR1 family inhibitors could enhance chemotherapy efficacy and improve outcomes in patients with PDAC. Future studies should focus on further elucidating the molecular pathways connecting Cldn1 and AKR proteins and exploring the therapeutic potential of Cldn1-targeted therapies in clinical settings.

## Figures and Tables

**Table 1:** Univariate and multivariate analyses of overall survival in patients with pancreatic adenocarcinoma.

	Univariate Analysis					Multivariate Analysis				
Variables	Hazard Ratio	95%CI (Lower)	95%CI (Upper)	*p*-Value		Hazard Ratio	95%CI (Lower)	95%CI (Upper)	*p*-Value	
Cldn1 positive	2.03	1.06	3.91	0.033	*	2.21	1.13	4.32	0.021	*
Male	0.74	0.42	1.29	0.284						
Age > 65	1.04	0.58	1.88	0.890						
ASA: 3	1.21	0.48	3.06	0.684						
BMI > 25	1.09	0.54	2.18	0.816						
Tumor location: Head	1.17	0.64	2.12	0.616						
Resectability: BR/UR	1.44	0.82	2.55	0.208						
Neoadjuvant treatment	1.14	0.63	2.06	0.655						
Poorly differentiated carcinoma	1.21	0.51	2.84	0.669						
Lymphatic invasion	1.38	0.77	2.48	0.275						
Vascular invasion	1.18	0.66	2.11	0.585						
Neural invasion	0.93	0.46	1.87	0.843						
Pleural invasion	0.98	0.50	1.92	0.955						
Portal vein invasion	0.90	0.45	1.80	0.761						
T-stage: pT3-4	1.74	0.89	3.40	0.108						
N-stage: pN1	1.98	1.08	3.63	0.027	*					
TNM stage: pStage III/IV	2.89	1.59	5.27	<0.001	**	2.85	1.50	5.42	0.001	**
High pre-operative CA19-9 (>37 U/mL)	1.35	0.76	2.43	0.308						
High post-operative CA19-9 (>37 U/mL)	3.66	1.96	6.85	<0.001	**	3.32	1.73	6.36	<0.001	**

* *p* < 0.05, ** *p* < 0.01.

**Table 2:** Downregulated proteins related to metabolic pathways of Cldn1-KO cells.

Accession	Protein Names	Gene Names	Ratio (KO-1/wt)	*p* Value (KO-1vs wt)	Ratio (KO-2/wt)	*p* Value (KO-2vs wt)
P47985	Cytochrome b-c1 complex subunit Rieske, mitochondrial	UQCRFS1	0.001	3.8 × 10^−5^	0.001	3.8 × 10^−5^
Q14697-2	Isoform 2 of Neutral alpha-glucosidase AB	GANAB	0.003	8.5 × 10^−6^	0.44	7.1 × 10^−3^
P52895	Aldo-keto reductase family 1 member C2	AKR1C2	0.06	2.4 × 10^−6^	0.01	8.7 × 10^−7^
P40261	Nicotinamide N-methyltransferase	NNMT	0.09	9.2 × 10^−11^	0.15	7.0 × 10^−11^
O00469-1	procollagen-lysine,2-oxoglutarate 5-dioxygenase 2	PLOD2	0.21	3.1 × 10^−3^	0.31	6.0 × 10^−3^
P42330	Aldo-keto reductase family 1 member C3	AKR1C3	0.37	1.4 × 10^−7^	0.19	9.2 × 10^−10^
P45954	Short/branched chain specific acyl-CoA dehydrogenase, mitochondrial	ACADSB	0.37	2.0 × 10^−2^	0.40	4.7 × 10^−2^
O75828	Carbonyl reductase [NADPH] 3	CBR3	0.40	7.4 × 10^−7^	0.39	1.5 × 10^−8^
Q02127	Dihydroorotate dehydrogenase (Quinone), mitochondrial	DHODH	0.46	3.0 × 10^−2^	0.31	2.6 × 10^−3^
P48163	NADP-dependent malic enzyme	ME1	0.49	2.6 × 10^−5^	0.34	4.1 × 10^−5^
P08243-1	Asparagine synthetase [glutamine-hydrolyzing]	ASNS	0.50	2.5 × 10^−8^	0.54	1.6 × 10^−7^
O14521	Succinate dehydrogenase [ubiquinone] cytochrome b small subunit, mitochondrial	SDHD	0.51	2.3 × 10^−4^	0.47	8.2 × 10^−5^
Q15125	3-beta-hydroxysteroid-Delta(8), Delta(7)-isomerase	EBP	0.54	9.6 × 10^−5^	0.60	5.7 × 10^−4^
Q01581	Hydroxymethylglutaryl-CoA synthase, cytoplasmic	HMGCS1	0.55	5.7 × 10^−6^	0.45	1.7 × 10^−3^
P15121	Aldose reductase	AKR1B1	0.56	1.2 × 10^−8^	0.31	3.0 × 10^−7^
Q9BWD1	Acetyl-CoA acetyltransferase, cytosolic	ACAT2	0.58	6.1 × 10^−4^	0.66	8.2 × 10^−4^
P52788-1	Spermine synthase	SMS	0.58	1.3 × 10^−4^	0.43	5.3 × 10^−7^
P30566	Adenylosuccinate lyase	ADSL	0.59	6.8 × 10^−6^	0.31	5.3 × 10^−8^
P09601	Heme oxygenase 1	HMOX1	0.59	6.5 × 10^−3^	0.13	1.7 × 10^−4^
P04181-1	Ornithine aminotransferase, mitochondrial	OAT	0.60	3.0 × 10^−4^	0.64	4.4 × 10^−4^
P15531	Nucleoside diphosphate kinase A	NME1	0.60	5.7 × 10^−4^	0.50	6.4 × 10^−4^
O00330-1	Pyruvate dehydrogenase protein X component, mitochondrial	PDHX	0.60	2.1 × 10^−6^	0.66	1.4 × 10^−3^
Q14693-1	Phosphatidate phosphatase LPIN1	LPIN1	0.61	2.8 × 10^−3^	0.39	2.7 × 10^−4^
Q96EM0	Trans-3-Hydroxy-L-proline dehydratase	L3HYPDH	0.64	1.9 × 10^−4^	0.31	1.1 × 10^−4^
P13674-1	Prolyl 4-hydroxylase subunit alpha-1	P4HA1	0.64	3.2 × 10^−5^	0.62	1.2 × 10^−5^
Q969N2	GPI transamidase component PIG-T	PIGT	0.65	3.3 × 10^−3^	0.44	3.5 × 10^−4^
P53701	Cytochrome c-type heme lyase	HCCS	0.65	8.1 × 10^−4^	0.38	4.2 × 10^−5^
Q7L5N7	Lysophosphatidylcholine acyltransferase 2	LPCAT2	0.65	2.3 × 10^−5^	0.58	7.8 × 10^−6^

## Data Availability

Data are contained within the article. The results are in part based upon data generated by the TCGA Research Network: https://www.cancer.gov/tcga (accessed on 1 February 2025), KM Plotter: https://pancreas.kmplot.com/ (accessed on 1 February 2025), and cBioPortal: https://www.cbioportal.org/ (accessed on 24 July 2023) [80,81,82,83,84].

## Supplementary Materials

The following supporting information can be downloaded at https://www.mdpi.com/article/10.3390/cancers17091469/s1: Figure S1: Cldn1 is expressed in less differentiated cancer cells and correlates with poor prognosis in patients with PDAC; Figure S2: AKR1 proteins correlated with Cldn1 expression; Text S1: Supplemental Figure Legends; Table S1: List of antibodies; Table S2: List of siRNAs; Table S3: Associations between claudin 1 expression and clinicopathologic features of PDAC; Table S4: Relationship between claudin 1 expression and prognosis of patients with pancreatic cancer in public databases; Table S5: Enriched GO terms and KEGG pathways assigned for downregulated proteins in *Cldn1-*KO cells; Table S6: Enriched GO terms assigned for upregulated proteins in Cldn1-KO cells; Data S1: Comprehensive shotgun proteome analysis between *Cldn1*-KO and wild-type cells.

## Abbreviations

The following abbreviations are used in this manuscript:

PDAC	pancreatic ductal adenocarcinoma
Cldn1	claudin-1
AKR	aldo-keto reductase
EMT	epithelial-mesenchymal transition
DD	dihydrodiol dehydrogenase
H-score	histological score
GO	Gene Ontology
KEGG	Kyoto Encyclopedia of Genes and Genomes
DAPI	4,6-diamidino-2-phenylindole
