# Supplementary material for: The Role of Claudin-1 in Enhancing Pancreatic Cancer Aggressiveness and Drug Resistance via Metabolic Pathway Modulation
Source: Cancers (Basel). 2025 Apr 27;17(9):1469. doi: 10.3390/cancers17091469 (PMC12070999; doi:10.3390/cancers17091469)
Supplement: Supplementary file 1 [file cancers-17-01469-s001.zip › Supplementary Table S1 20250407.docx]

**Supplemental Table 1. List of antibodies.**

| Antibodies | Type | Supplier | Catalog # |
| --- | --- | --- | --- |
| Primary antibodies | | | |
| anti-claudin-1 | Rabbit polyclonal | Thermo Fischer Scientific | 51-9000 |
| anti-claudin-1 | Mouse monoclonal | Santa Cruz | Sc-166338 |
| anti-occludin | Rabbit polyclonal | Thermo Fischer Scientific | 71-1500 |
| anti-actin | Rabbit polyclonal | Sigma-Aldrich | A2066 |
| anti-Ki67 | Mouse monoclonal | DAKO | M7240 |
| anti-AKR1B1 | Rabbit polyclonal | ABclonal | A13944 |
| anti-AKR1B1 | Mouse monoclonal | Santa Cruz | sc-373953 |
| anti-AKR1C2 | Rabbit polyclonal | ABclonal | A1048 |
| anti-AKR1C3 | Rabbit polyclonal | ABclonal | A13568 |
| anti-DD | Mouse monoclonal | Santa Cruz | sc-166297 |
| Isotype control | Rabbit polyclonal | Cell Signaling Technology | #2729 |
| Second antibodies | | | |
| anti-mouse IgG |  | DAKO | P0161 |
| anti-rabbit IgG |  | DAKO | P0448 |
| DyLight® 488 (green)-conjugated anti-rabbit IgG |  | Bethyl Laboratories | A120-101D2 |
| DyLight® 594 (red)-conjugated anti-mouse IgG |  | Bethyl Laboratories | A90-116D4 |
| DAPI |  | Dojindo | 340-07971 |

ABclonal (Tokyo, Japan); Cell Signaling Technology (Danvers, MA, USA); DAKO (Glostrup, Denmark); Sigma-Aldrich (St. Louis, MO, USA); Santa Cruz (Dallas, TX, USA); Thermo Fischer Scientific (Waltham, MA, USA), Bethyl Laboratories (Waltham, MA, USA), Dojindo (Kumamoto, Japan).
