# Supplementary material for: The Role of Claudin-1 in Enhancing Pancreatic Cancer Aggressiveness and Drug Resistance via Metabolic Pathway Modulation
Source: Cancers (Basel). 2025 Apr 27;17(9):1469. doi: 10.3390/cancers17091469 (PMC12070999; doi:10.3390/cancers17091469)
Supplement: Supplementary file 1 [file cancers-17-01469-s001.zip › Supplementary Table S2.docx]

**Supplemental Table 2. List of siRNAs.**

| Name of siRNA | Gene | Supplier | Sequences of siRNA (sense, 5’-3’) |
| --- | --- | --- | --- |
| anti-*AKR1C2*-1 | *AKR1C2* | Life technologies | CGGCCGGAAAAGAAAGACA |
| anti-*AKR1C2*-2 | *AKR1C2* | Merck | GCGAUAUUUGACCCUUGAU |
| anti-*AKR1C3*-1 | *AKR1C3* | Life technologies | GAAUGUCAUCCGUAUUUCA |
| anti-*AKR1C3-*1 | *AKR1C3* | Merck | CUUAUUCAUUCUCCAAUGU |
| anti-*AKR1B1*-1 | *AKR1B1* | Life technologies | AGCCUGCAGUUAACCAGAU |
| anti-*AKR1B1*-2 | *AKR1B1* | Merck | CAAACCUGGCUUGAAGUAU |

life technologies (Carlsbad, CA); Merck (Darmstadt, Germany)
