# Supplementary material for: The Role of Claudin-1 in Enhancing Pancreatic Cancer Aggressiveness and Drug Resistance via Metabolic Pathway Modulation
Source: Cancers (Basel). 2025 Apr 27;17(9):1469. doi: 10.3390/cancers17091469 (PMC12070999; doi:10.3390/cancers17091469)
Supplement: Supplementary file 1 [file cancers-17-01469-s001.zip › Supplementary Table S3.docx]

| Variables | Cldn1 negative (n=26) | Cldn1 positive (n=51) | p-value |
| --- | --- | --- | --- |
| Age |  |  |  |
| <65 | 13 (50.0%) | 14 (27.5%) | 0.076 |
| >65 | 13 (50.0%) | 37 (72.5%) |  |
| Gender |  |  |  |
| Female | 11 (42.3%) | 24 (47.1%) | 0.81 |
| Male | 15 (57.7%) | 27 (52.9%) |  |
| ASA |  |  |  |
| 0-2 | 24 (92.3%) | 45 (88.2%) | 0.71 |
| 3 | 2 (7.7%) | 6 (11.8%) |  |
| BMI |  |  |  |
| <25 | 23 (88.5%) | 40 (78.4%) | 0.36 |
| >25 | 3 (11.5%) | 11 (21.6%) |  |
| Tumor location |  |  |  |
| Head | 10 (38.5%) | 17 (33.3%) | 0.80 |
| Body/Tail | 16 (61.5%) | 34 (66.7%) |  |
| Resectability |  |  |  |
| R | 17 (65.4%) | 32 (62.7%) | 1 |
| BR/UR | 9 (34.6%) | 19 (37.3%) |  |
| Neoadjuvant treatment |  |  |  |
| No | 17 (65.4%) | 36 (70.6%) | 0.80 |
| Yes | 9 (34.6%) | 15 (29.4%) |  |
| Poorly differentiated carcinoma |  |  |  |
| Negative | 24 (92.3%) | 44 (86.3%) | 0.71 |
| Positive | 2 (7.7%) | 7 (13.7%) |  |
| Lymphatic invasion |  |  |  |
| Negative | 8 (30.8%) | 22 (43.1%) | 0.33 |
| Positive | 18 (69.2%) | 29 (56.9%) |  |
| Vascular invasion |  |  |  |
| Negative | 7 (26.9%) | 21 (41.2%) | 0.32 |
| Positive | 19 (73.1%) | 30 (58.8%) |  |
| Neural invasion |  |  |  |
| Negative | 7 (26.9%) | 7 (13.7%) | 0.21 |
| Positive | 19 (73.1%) | 44 (86.3%) |  |
| Portal vein invasion |  |  |  |
| Negative | 19 (73.1%) | 41 (80.4%) | 0.56 |
| Positive | 7 (26.9%) | 10 (19.6%) |  |
| T-stage |  |  |  |
| pT1-2 | 22 (84.6%) | 39 (76.5%) | 0.56 |
| pT3-4 | 4 (15.4%) | 12 (23.5%) |  |
| N-stage |  |  |  |
| pN0 | 8 (30.8%) | 22 (43.1%) | 0.33 |
| pN1 | 18 (69.2%) | 29 (56.9%) |  |
| TNM-stage |  |  |  |
| pStage I/II | 17 (65.4%) | 39 (76.5%) | 0.42 |
| pStage III/IV | 9 (34.6%) | 12 (23.5%) |  |
| Pre-operative CA19-9 |  |  |  |
| Within normal | 13 (50.0%) | 19 (37.3%) | 0.28 |
| Above normal (>37 U/mL) | 13 (50.0%) | 32 (62.7%) |  |
| Post-operative CA19-9 |  |  |  |
| Within normal | 4 (15.4%) | 14 (27.5%) | 0.26 |
| Above normal (>37 U/mL) | 22 (84.6%) | 33 (64.7%) |  |

Supplementary Table 3. Associations between claudin 1 expression and clinicopathologic features of PDAC.
