# Supplementary material for: The Role of Claudin-1 in Enhancing Pancreatic Cancer Aggressiveness and Drug Resistance via Metabolic Pathway Modulation
Source: Cancers (Basel). 2025 Apr 27;17(9):1469. doi: 10.3390/cancers17091469 (PMC12070999; doi:10.3390/cancers17091469)
Supplement: Supplementary file 1 [file cancers-17-01469-s001.zip › Supplementary Table S4.docx]

|  | High claudin 1 expression | HR (95%CI) | P-value |  | Reference |
| --- | --- | --- | --- | --- | --- |
| OS | unfavorable |  | 5.2 × 10^-4^ | Median survival: 15.87vs 20.2 m | 30 |
| OS | unfavorable | 1.36 (1.17-1.59) | 7.38 × 10^-5^ | FDR: 0.00142 | 31 |
| DFS | unfavorable | 1.82 (1.41-2.36) | 5.96 × 10^-6^ | FDR: 1.03×10^-4^ |  |

Supplemental Table 4: Relationship between claudin 1 expression and prognosis of patients with pancreatic cancer in public databases.

OS: overall survival; DFS: disease-free survival
