# Supplementary material for: The Role of Claudin-1 in Enhancing Pancreatic Cancer Aggressiveness and Drug Resistance via Metabolic Pathway Modulation
Source: Cancers (Basel). 2025 Apr 27;17(9):1469. doi: 10.3390/cancers17091469 (PMC12070999; doi:10.3390/cancers17091469)
Supplement: Supplementary file 1 [file cancers-17-01469-s001.zip › Supplementary Table S5.docx]

|  | Term | Count | Fold Enrichment | FDR |
| --- | --- | --- | --- | --- |
| **Biological process** | | | | |
| GO:0015670 | carbon dioxide transport | 4 | 47.02 | 2.2E-02 |
| GO:0015671 | oxygen transport | 4 | 41.15 | 2.2E-02 |
| GO:0042744 | hydrogen peroxide catabolic process | 5 | 29.39 | 1.8E-02 |
| GO:0098869 | cellular oxidant detoxification | 6 | 12.99 | 2.2E-02 |
| **Cellular component** | | | | |
| GO:0031838 | haptoglobin-hemoglobin complex | 4 | 52.62 | 2.8E-03 |
| GO:0005833 | hemoglobin complex | 4 | 48.86 | 2.8E-03 |
| GO:0042613 | MHC class II protein complex | 4 | 26.31 | 1.6E-02 |
| GO:0098553 | lumenal side of endoplasmic reticulum membrane | 4 | 23.59 | 1.9E-02 |
| GO:0030658 | transport vesicle membrane | 4 | 15.91 | 4.7E-02 |
| GO:0012507 | ER to Golgi transport vesicle membrane | 4 | 12.00 | 9.4E-02 |
| GO:0005739 | mitochondrion | 23 | 2.76 | 2.3E-03 |
| GO:0070062 | extracellular exosome | 29 | 2.21 | 2.8E-03 |
| GO:0005829 | cytosol | 64 | 1.98 | 3.6E-07 |
| GO:0005737 | cytoplasm | 49 | 1.49 | 4.2E-02 |
| **Molecular function** | | | | |
| GO:0043177 | organic acid binding | 4 | 56.75 | 9.3E-03 |
| GO:0016655 | oxidoreductase activity, acting on NAD(P)H,  quinone or similar compound as acceptor | 3 | 52.02 | 6.7E-02 |
| GO:0031720 | haptoglobin binding | 3 | 46.82 | 6.7E-02 |
| GO:0005344 | oxygen transporter activity | 4 | 39.01 | 1.4E-02 |
| GO:0004032 | alditol:NADP+ 1-oxidoreductase activity | 3 | 36.01 | 9.2E-02 |
| GO:0032395 | MHC class II receptor activity | 3 | 36.01 | 9.2E-02 |
| GO:0023026 | MHC class II protein complex binding | 4 | 23.12 | 5.1E-02 |
| GO:0019825 | oxygen binding | 4 | 16.87 | 6.7E-02 |
| GO:0042605 | peptide antigen binding | 4 | 13.00 | 9.9E-02 |
| GO:0020037 | heme binding | 8 | 8.05 | 9.3E-03 |
| GO:0005515 | protein binding | 97 | 1.19 | 6.7E-02 |
| **KEGG pathway** | | | | |
| hsa01100 | Metabolic pathways | 28 | 2.31 | 2.3E-03 |

Supplementary Table 5. Enriched GO terms and KEGG pathways assigned for downregulated (ratio < 0.666, *P* < .05 and FDR<.05 vs Wt) proteins in *Cldn1*-KO cells.

GO: Gene Ontology; KEGG: Kyoto Encyclopedia of Genes and Genomes; FDR: False discovery rate.
