# Supplementary material for: The Role of Claudin-1 in Enhancing Pancreatic Cancer Aggressiveness and Drug Resistance via Metabolic Pathway Modulation
Source: Cancers (Basel). 2025 Apr 27;17(9):1469. doi: 10.3390/cancers17091469 (PMC12070999; doi:10.3390/cancers17091469)
Supplement: Supplementary file 1 [file cancers-17-01469-s001.zip › Supplementary Table S6.docx]

|  | Term | Count | Fold Enrichment | FDR |
| --- | --- | --- | --- | --- |
| **Biological process** | |  |  |  |
| GO:1901254 | positive regulation of intracellular transport of viral material | 3 | 95.33 | 9.9E-02 |
| GO:0045947 | negative regulation of translational initiation | 5 | 22.70 | 4.6E-02 |
| GO:0006417 | regulation of translation | 8 | 7.78 | 4.6E-02 |
| GO:0043065 | positive regulation of apoptotic process | 13 | 3.86 | 6.0E-02 |
| **Cellular component** | |  |  |  |
| GO:1990812 | growth cone filopodium | 3 | 73.64 | 2.5E-02 |
| GO:1902737 | dendritic filopodium | 4 | 56.11 | 3.5E-03 |
| GO:0044326 | dendritic spine neck | 3 | 49.09 | 4.5E-02 |
| GO:0071598 | neuronal ribonucleoprotein granule | 3 | 42.08 | 5.7E-02 |
| GO:0043232 | intracellular non-membrane-bounded organelle | 4 | 17.85 | 4.5E-02 |
| GO:0000775 | chromosome, centromeric region | 7 | 10.91 | 3.5E-03 |
| GO:0043679 | axon terminus | 5 | 8.05 | 8.0E-02 |
| GO:0035578 | azurophil granule lumen | 6 | 6.47 | 5.9E-02 |
| GO:1904813 | ficolin-1-rich granule lumen | 8 | 6.28 | 1.4E-02 |
| GO:0005694 | chromosome | 10 | 3.82 | 4.5E-02 |
| GO:0005925 | focal adhesion | 12 | 2.75 | 9.7E-02 |
| GO:0070062 | extracellular exosome | 42 | 1.84 | 7.0E-03 |
| GO:0005829 | cytosol | 98 | 1.74 | 2.9E-07 |
| GO:0005737 | cytoplasm | 96 | 1.67 | 2.2E-06 |
| GO:0005654 | nucleoplasm | 64 | 1.58 | 6.5E-03 |
| **Molecular function** | |  |  |  |
| GO:0033592 | RNA strand annealing activity | 3 | 55.81 | 6.2E-02 |
| GO:0086083 | cell adhesive protein binding involved in bundle of  His cell-Purkinje myocyte communication | 3 | 55.81 | 6.2E-02 |
| GO:0019215 | intermediate filament binding | 3 | 39.87 | 9.4E-02 |
| GO:0002151 | G-quadruplex RNA binding | 4 | 37.21 | 1.4E-02 |
| GO:0044325 | ion channel binding | 9 | 5.90 | 1.4E-02 |
| GO:0008017 | microtubule binding | 13 | 4.45 | 7.5E-03 |
| GO:0045296 | cadherin binding | 11 | 3.20 | 9.4E-02 |
| GO:0042803 | protein homodimerization activity | 20 | 2.50 | 3.2E-02 |
| GO:0042802 | identical protein binding | 33 | 1.77 | 7.9E-02 |
| GO:0005515 | protein binding | 167 | 1.22 | 5.5E-04 |

Supplementary Table 6. Enriched GO terms assigned for upregulated (ratio > 1.5, *P* < .05 and FDR<.05 vs Wt) proteins in CLDN1-KO cells. Enrichment analysis showed no significant KEGG Pathways. GO: Gene Ontology; KEGG: Kyoto Encyclopedia of Genes and Genomes; FDR: False discovery rate.
