# Supplementary material for: The Role of Claudin-1 in Enhancing Pancreatic Cancer Aggressiveness and Drug Resistance via Metabolic Pathway Modulation
Source: Cancers (Basel). 2025 Apr 27;17(9):1469. doi: 10.3390/cancers17091469 (PMC12070999; doi:10.3390/cancers17091469)
Supplement: Supplementary file 1 [file cancers-17-01469-s001.zip › Supplementary text S1.docx]

**Supplemental Figure Legends**

**Supplemental Figure 1**. Claudin-1 (Cldn1) is expressed in less differentiated cancer cells and correlates with poor prognosis in patients with pancreatic ductal adenocarcinoma (PDAC). A: Immunohistochemistry for Cldn1 in normal ducts and low- and high-grade pancreatic intraepithelial neoplasia (L- and H-PanIN). Cldn1 was expressed on the plasma membrane in normal pancreatic ductal epithelium and L-PanIN, whereas it was highly expressed in the cytoplasm of H-PanIN. B: Kaplan–Meier curves for survival rates of patients with PDAC divided by Cldn1 expression levels. These graphs were created using public databases (KM Plotter; https://pancreas.kmplot.com/). The left graph shows the overall survival rate, and the right graph shows the disease-free survival rate. The log-rank test and Cox proportional hazards model analysis showed a significant difference in the survival rates between the two groups. C and D: Immunohistochemistry of Cldn1 in PDAC tissues. Cldn1 was highly expressed in the metastatic lymph nodes, nerve (C), and invasive front (D). Arrows indicate infiltrated nerve tissue (C). Squares in the left panel (magnification ×40) indicate the locations in the middle and right panels (D; magnification ×400). E: Overall survival days for each group classified by the H-score. There was a trend of decreasing survival days with increasing H-scores.

**Supplemental Figure 2**. Aldo-keto reductase 1 (AKR1) proteins correlated with Cldn1 expression. A: Correlation between Cldn1 and AKR1 mRNA expression. These graphs were created based on the results of combining the datasets from five public databases (cBioPortal; https://www.cbioportal.org/) [65–69]. B: WST-8 assay of PK45H cells transfected with siRNAs specific for AKR1C2, AKR1C3, and AKR1B1 and negative control (NC) using etoposide, doxorubicin, and daunorubicin. In this experiment, we used siRNAs different from those shown in Fig. 4E. C: WST-8 cell proliferation assay of AKR1C2-, AKR1C3-, and AKR1B1-knockdown clones and NC. In this experiment, we used siRNAs different from those shown in Fig. 5A. D and E: Wound healing (D) and migration and invasion assay using a double chamber (E) of NC and each KD clone. Right-sided graphs show the quantification of each assay. F: Immunohistochemistry of Cldn1, dihydrodiol dehydrogenase, and AKR1B1 in the same area. The upper panel shows pancreatic cancer cells invading the nerve (arrows); magnification of upper panels ×200; magnification of lower panels ×400). Bar: 100um. Graphs represent mean ± SD. *P<0.05 vs. NC.
