# Supplementary figures and images for: The Role of Claudin-1 in Enhancing Pancreatic Cancer Aggressiveness and Drug Resistance via Metabolic Pathway Modulation
Source: Cancers (Basel). 2025 Apr 27;17(9):1469. doi: 10.3390/cancers17091469 (PMC12070999; doi:10.3390/cancers17091469)

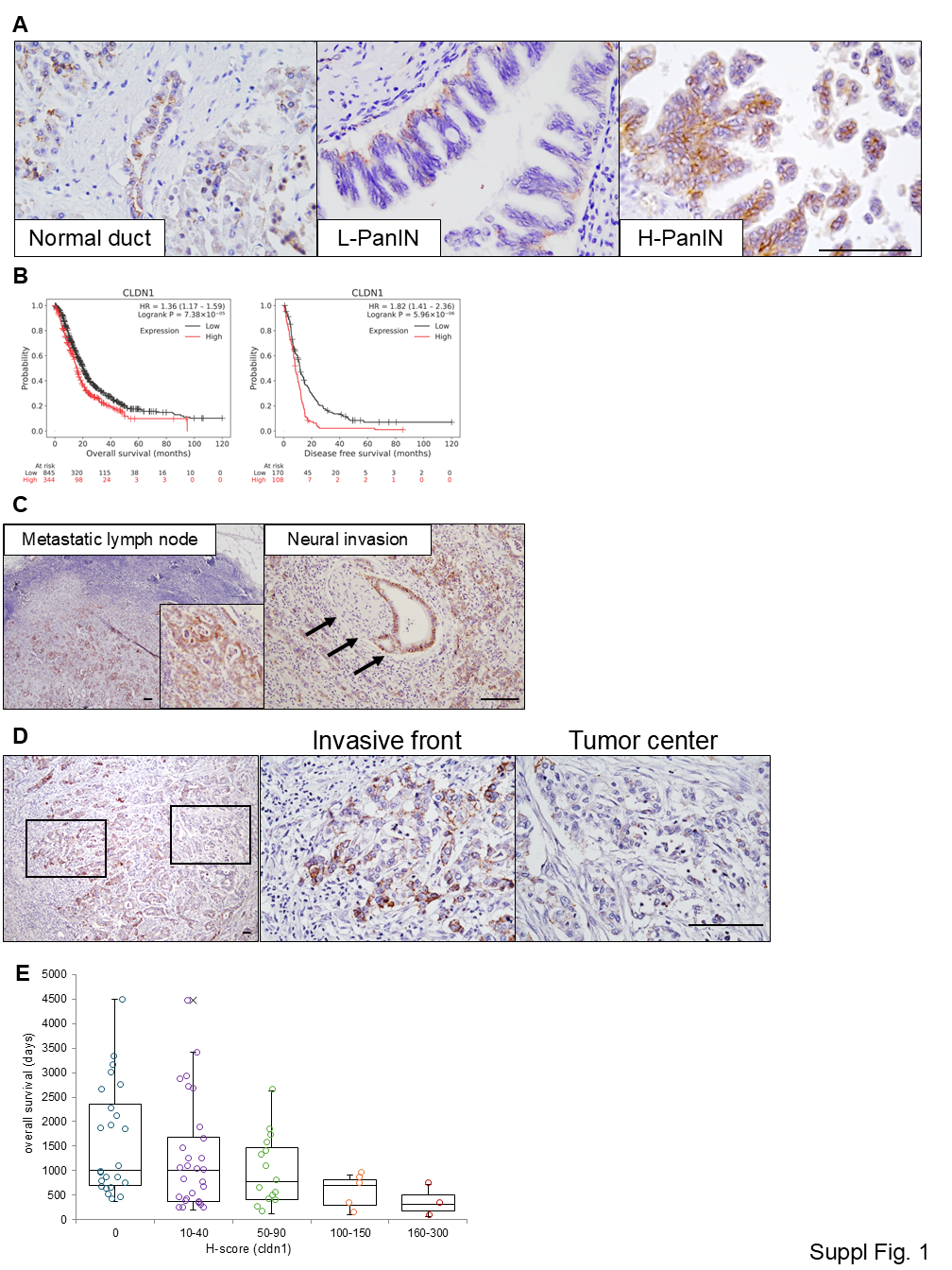

Supplement: Supplementary file 1 [file cancers-17-01469-s001.zip › Supplementary Fig. S1.tif]

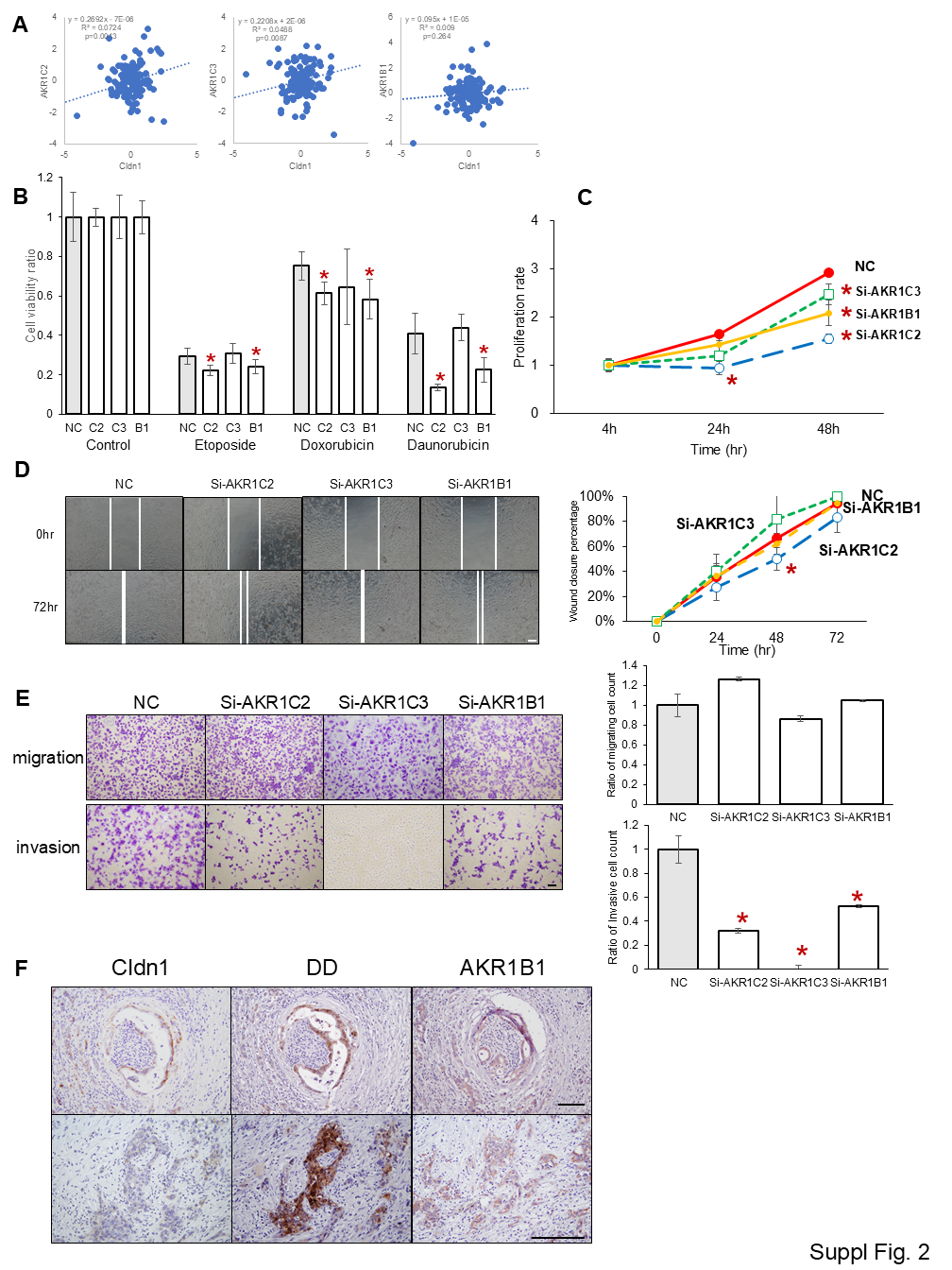

Supplement: Supplementary file 1 [file cancers-17-01469-s001.zip › Supplementary Fig. S2.tif]
